# Knowledge and determinants regarding tuberculosis among medical students in Hunan, China: a cross-sectional study

**DOI:** 10.1186/s12889-018-5636-x

**Published:** 2018-06-13

**Authors:** Yangjiang Ou, Zhenzhou Luo, Jinsong Mou, Hui Ming, Xiang Wang, Shipeng Yan, Aichun Tan

**Affiliations:** 1 School Key Discipline of Nutrition and Food Hygiene, Public Health School, Changsha Medical University, Changsha, People’s Republic of China; 2 Department of Dermatology and Venereology, Shenzhen Nanshan Center for Chronic Disease Control, Shenzhen, People’s Republic of China; 3 Shenzhen Pingshan Maternal and Child Health Hospital, Shenzhen, 518122 People’s Republic of China; 4 Hunan Institute for Tuberculosis Control and Hunan Chest Hospital, Changsha, People’s Republic of China; 5 Yongzhou Center for Disease Control and Prevention, Yongzhou, People’s Republic of China; 6 Hunan Province Cancer Hospital, Changsha, People’s Republic of China; 7 Xiangya School of Public Health, Central South University, Changsha, People’s Republic of China

**Keywords:** Knowledge, Determinants, Tuberculosis, Medical students

## Abstract

**Background:**

Tuberculosis (TB) is one of the most common infectious diseases worldwide. Insufficient TB knowledge may increase the risk of contracting the disease among medical students. The purpose of this study was to assess the level of TB knowledge and analyse related determinants among medical students.

**Methods:**

A cross-sectional study was performed among final-year medical students from three main undergraduate medical universities in Hunan Province. TB knowledge, attitude and practice were assessed using a questionnaire. A t-test and multiple linear regression analysis were conducted to explore the association between TB knowledge and influencing factors.

**Results:**

The total mean percentage of correct answers for TB knowledge was 44.4% (SD 13.5%), including 52.5% (SD 16.8%) for epidemiology and prevention, 35.7% (SD 16.1%) for diagnosis, and 47.5% (SD 22.7%) for treatment. Medical students who reported observing at least one TB case and an X-ray of a TB patient had a higher percentage of correct answers for epidemiology and prevention (54.4% vs 43.9%, *p* < 0.001; 54.3% vs 42.1%, *p* < 0.001), diagnosis (37.2% vs 29.0%, *p* < 0.001; 37.1% vs 27.5%, *p* < 0.001), treatment (50.0% vs 36.0%, *p* < 0.001; 49.5% vs 35.7%, *p* < 0.001) and total score (46.2% vs 36.2, *p* < 0.001; 46.0% vs 34.7%, *p* < 0.001). Older medical students (≥23 years) had greater knowledge than younger medical students (< 23 years) regarding diagnosis (37.2% vs 31.7%, *p* < 0.001). The multivariable linear regression analysis determined an association between observing at least one TB case and an X-ray of a TB patient and greater knowledge of epidemiology and prevention (β = 5.6, 95% CI: 2.3, 8.9; β = 8.2, 95% CI: 4.6, 11.8), diagnosis (β = 3.9, 95% CI: 0.8, 7.1; β = 5.7, 95% CI: 2.2, 9.2) and treatment (β = 10.1, 95% CI: 5.6, 14.5; β = 7.0, 95% CI: 2.2, 11.8) and a higher total score (β = 5.5, 95% CI: 2.9, 8.1; β = 6.6, 95% CI: 3.8, 9.5). Moreover, an older age (≥23 years) was associated with more accurate knowledge of diagnosis (β = 3.9, 95% CI: 1.8, 6.1) and a higher total score (β = 2.8, 95% CI: 1.1, 4.6).

**Conclusion:**

Poor TB knowledge was observed among medical students, which implied a need to innovate our current infectious disease curriculum to promote TB knowledge and practices among medical students.

## Background

Tuberculosis (TB) is one of the most common infectious diseases worldwide and continues to be a major public health problem for low and middle-income countries. Several effective strategies have been implemented by the World Health Organization (WHO) to prevent and control the disease, including the directly observed treatment, short-course (DOTS) and stop TB strategies [1, 2]. The Millennium Development Goals (MDGs) [3], which were developed to halt and reverse the TB incidence by 2015, have been achieved, with a total reduction of 18% worldwide, and the death rate is half that reported in 1990 [4]. Despite these gains, the epidemic remains serious. The Global TB Report 2017 [5] published by the WHO reported that, although the fight against TB was paying off, 1.674 million people died from TB in 2016, and the disease ranked as the leading cause of death from a single infectious agent above HIV/AIDS. The report also showed that China, India and Indonesia alone accounted for 45% of global cases in 2016 and that China had a high TB burden even though the country had achieved the MDGs to reduce the prevalence of smear-positive TB by 50% in 2010 [6].

TB trends are influenced by control programmes as well as by biological, social and economic factors [7]. Undoubtedly, a lack of knowledge regarding TB among health care workers may contribute to an increased risk of developing the disease [8, 9]. A study of Chinese TB patients showed that a TB diagnostic delay was related to health facility staff factors, including inadequate TB knowledge, inability to prescribe a smear test for suspected TB cases, inability to refer suspected TB cases to county TB dispensaries or designated hospitals for TB care and misdiagnosis [10]. In this context, TB knowledge among medical students is particularly important; thus, undergraduate training regarding TB should be strengthened, because students may face significant exposures and consequently have the highest risk of infection or disease [11]. Furthermore, because medical students are potential future physicians and leaders, these students need to understand the epidemiology, determinants, screening and management of TB to promote effective prevention, early diagnosis, and successful treatment [12].

However, several studies [13–15] have reported that the TB knowledge of undergraduate medical students is insufficient. Medical colleges play an important role in training and shaping the attitudes of the future generations of medical practitioners [14]. Although TB health education in schools was emphasized at all times in China, researchers [16, 17] found that TB knowledge and practices among medical students were generally inadequate. A dearth of data regarding TB knowledge exists among medical students in China. Moreover, Hunan Province reported a notifiable TB incidence rate of 83.0 per 100,000 populations, which was the fourth highest incidence in China [18]. With this background, this study was conducted to survey 1088 preclinical undergraduate students from three medical universities in Hunan Province with the objective of assessing TB knowledge among final-year medical students and analysing the related determinants. Our findings can serve as baseline information for TB knowledge and help improve future TB prevention and control strategies in the region.

## Methods

### Study population

A cross-sectional study was conducted among three main undergraduate medical universities located in Changsha, which is the capital of Hunan Province, China, from March to June of 2016. Final-year medical students were recruited, because they had taken the infectious disease course prior to this investigation.

### The questionnaire

The questionnaire was designed based on the report of Laurenti [19] and adjusted appropriately. The questionnaire focused on two dimensions: attitudes and experiences and knowledge of epidemiology and prevention, diagnosis and treatment. Each question had five possible answers, of which only one was correct. The students were asked to circle the most appropriate answer independently without receiving any help completing the questionnaire. All students participated on a voluntary basis and were not remunerated for their contribution.

### Data analysis

The information was recorded in a database using EpiData 3.0 and analysed using SPSS 18.0. The data are reported as percentages. The total score for all questions and separate scores for epidemiology and prevention, diagnosis, and treatment were calculated. In addition, the mean percentage scores in different subpopulations were calculated based on demographic variables, experiences, and attitudes. Descriptive statistics and t-tests were used to analyse differences in different subpopulations. A multivariable linear regression analysis was performed to analyse the determinants associated with epidemiology and prevention, diagnosis, and treatment on TB knowledge.

## Results

### General characteristics of the participants

A total of 1200 undergraduate medical university students were surveyed, of whom 112 were excluded due to incomplete responses or logical errors. A total of 1088 valid questionnaires were received, for a response rate of 90.7%. Table 1 shows the characteristics of the participants. A total of 435 (40.0%) and 653 (60.0%) of the respondents were male and female, respectively, resulting in a gender ratio of 1:1.5. The mean age was 23 ± 0.92 years, and the ages ranged between 20 and 25 years. Only 160 (14.7%) of the students had received a PPD (purified protein derivative) test, and only 77 (10%) had performed at least one PPD test. More than 80% of the students had observed at least one case of TB and an X-ray of a TB patient (*n* = 891, 81.9% and *n* = 929, 85.4%, respectively). Approximately one-fourth (*n* = 293, 26.9%) of the students were aware of being at risk for TB infection.

**Table 1:** Demographic characteristics, experiences and attitudes of participants

Characteristics of participants	Number	Percent
Demographic		
Gender		
Male	435	40.0
Female	653	60.0
Age (Std. Dev.)	23(0.92)	
Experiences and Attitudes		
Received PPD test		
Yes	160	14.7
No	928	85.3
Performed at least one PPD test		
Yes	77	7.1
No	1011	92.9
Observed at least one TB case		
Yes	891	81.9
No	197	18.1
Observed at least one X-ray of TB case		
Yes	929	85.4
No	159	14.6
I’m at risk for TB		
Yes	293	26.9
No	795	73.1

### Knowledge about the epidemiology and prevention, diagnosis, and treatment of TB

The total mean percentage of correct answers for TB knowledge was 44.4% (SD 13.5%). The mean percentages of correct answers were 52.5% (SD 16.8%) for epidemiology and prevention, 35.7% (SD 16.1%) for diagnosis, and 47.5% (SD 22.7%) for treatment. In the epidemiology and prevention dimension (Table 2), the mean score for “BCG (bacille Calmette-Guerin) is a vaccine for TB” was relatively high at 90.6%, whereas the mean score for “The *Mycobacterium avium* complex is the most frequent aetiologic agent of TB” was lowest at 6.4%.

**Table 2:** Mean percentage of correct answers in epidemiology and prevention

Correct answers	Mean percentage (%)
BCG is a vaccine for TB	90.6
TB transmitted via airborne	89.7
Higher incidence and delay in diagnosis of TB related to disequalities, Socio-economic issues and low resources of native country	67.7
Prevalence of TB in India and China is highest	64.9
Health operators, immigrants, elderly, children, immunosuppressed patients are at more risk of TB	63.5
Masks are necessary for entering a room where an open TB patient stays	55.9
The current TB vaccine is live attenuated vaccine	52.2
Patients with both latent TB infection and HIV infection will increase the risk for developing active TB	44.8
TB vaccination in low prevalence populations is not useful in controlling the spread of TB	41.2
Isolate an open pulmonary TB patient using a negative pressure room	38.7
HIV is strongly related with the TB bacillus	37.7
The prevalence of TB in China is currently 60–70 cases per 100,000 people	28.9
Mycobacterium Avium Complex is the most frequent aetiologic agent of TB	6.4

In the diagnosis dimension (Table 3), the mean score for “A sputum test is necessary to detect suspected pulmonary TB” was highest at 69.9%, whereas the mean score for “Koch bacillus identification is always performed using a Ziehl-Neelsen stain” was lowest at 8.8%.

**Table 3:** Mean percentage of correct answers in diagnosis and treatment

Correct answers	Mean percentage (%)
Diagnosis	
Sputum test is necessary to detect suspected pulmonary TB	69.9
Chest x-ray is recommended when someone has a positive tuberculin skin test	63.4
Lung, brain, kidney could be affected by M. Tuberculosis	53.7
Positive intradermal reaction test indicates increased sensitization to drugs	50.2
First contact with bacteria causes a primary complex	47.9
Patients and health operators which come in contact with a TB patient should receive PPD test	37.4
PPD test is positive from an induration diameter of 5 mm	36.9
PPD test is an intradermal injection	31.3
The immune response to TB is cell mediated	27.4
“Quantiferon TB gold” test can differentiate infection from other reasons for testing positive	22.7
It is an expression of immune response to mycobacterial antigens which is not a limit of tuberculin skin test.	20.6
After 5 weeks, the primary infection does tuberculin test result will become positive	19.5
Tine test is a multiple puncture test	10.1
Koch bacillus identification is always performed through Ziehl–Neelsen stain	8.8
Treatment	
Penicillin G is not useful for TB treatment	74.9
In case of positive PPD test in an exposed health operator, he/she should be assessed for further investigations and for prophylactic treatment with isoniazid	61.3
Prophylactic treatment with isoniazid is implemented to close contacts resulted negative to the test	46.6
Consider an active TB woman in homecare therapy who has recently discovered to be pregnant, she should be informed that tuberculosis does not increase the risk of miscarriage and treatment should be changed because there is a potential risk of toxicity for the child	35.7
TB patient supposed to be hospitalized for an effective treatment every 7–10 days	19.2

In the treatment dimension (Table 3), the mean score for “Penicillin G is not useful for TB treatment” was highest at 74.6%, whereas the mean score for “TB patients are supposed to be hospitalized for effective treatment every 7-10 days” was lowest at 19.2%.

### Determinants of TB knowledge

Table 4 shows the lack of an association between gender and the mean percentage of correct answers. The older medical students (≥23 years) had more knowledge than the younger medical students (< 23 years) regarding epidemiology and prevention (53.6% vs 49.6%, *p* = 0.001), diagnosis (37.2% vs 31.7%, *p* < 0.001) and treatment (49.0% vs 43.5%, *p* < 0.001) and higher total scores (45.7% vs 40.8%, *p* < 0.001). Medical students who reported receiving a PPD test had a higher percentage of correct answers in the total score (46.3% vs 44.0%, *p* = 0.047); conversely, no association was found between whether medical students reported performing at least one PPD test and the mean percentage of correct answers. Medical students who reported observing at least one TB case and one X-ray for a TB case had higher percentages of correct answers for epidemiology and prevention (54.4% vs 43.9%, *p* < 0.001; 54.3% vs 42.1%, *p* < 0.001), diagnosis (37.2% vs 29.0%, *p* < 0.001; 37.1% vs 27.5%, *p* < 0.001), and treatment (50.0% vs 36.0%, *p* < 0.001; 49.5% vs 35.7%, *p* < 0.001) and higher total scores (46.2% vs 36.2%, *p* < 0.001; 46.0% vs 34.7%, *p* < 0.001). Medical students who were aware of themselves being at risk for TB reported a similar percentage of correct answers with the other students.

**Table 4:** Mean percentage of correct answer by demographic characteristics, experiences and attitudes of participants

	Epidemiology and prevention		Diagnosis		Treatment		Total	
	Mean score	*P*-value	Mean score	*P*-value	Mean score	*P*-value	Mean score	*P*-value
Gender								
Male	51.8	0.316	35.8	0.801	48.9	0.092	44.4	0.947
Female	52.9		35.6		46.6		44.3	
Age								
< 23	49.6	0.001	31.7	< 0.001	43.5	< 0.001	40.8	< 0.001
≥ 23	53.6		37.2		49.0		45.7	
Received PPD test								
Yes	54.2	0.153	38.0	0.070	47.5	0.990	46.3	0.047
No	52.2		35.3		47.5		44.0	
Performed at least one PPD test								
Yes	53.9	0.426	36.8	0.523	47.5	0.990	45.5	0.462
No	52.4		35.6		47.5		44.3	
Observed at least one TB case								
Yes	54.4	< 0.001	37.2	< 0.001	50.0	< 0.001	46.2	< 0.001
No	43.9		29.0		36.0		36.2	
Observed at least one X-ray of TB case								
Yes	54.3	< 0.001	37.1	< 0.001	49.5	< 0.001	46.0	< 0.001
No	42.1		27.5		35.7		34.7	
I’m at risk for TB								
Yes	53.2	0.395	35.1	0.429	45.5	0.072	44.0	0.651
No	52.2		35.9		48.3		44.5	

The multivariable linear regression analysis (Table 5) identified an association between observing at least one TB case and an X-ray of a TB case and greater knowledge regarding epidemiology and prevention (β = 5.6, 95% CI: 2.4, 8.9; β = 8.2, 95% CI: 4.6, 11.8), diagnosis (β = 3.9, 95% CI: 0.8, 7.1; β = 5.7, 95% CI: 2.2, 9.2), treatment (β = 10.1, 95% CI: 5.6, 14.5; β = 7.0, 95% CI: 2.2, 11.8) and total scores (β = 5.5, 95% CI: 2.9, 8.1; β = 6.6, 95% CI: 3.8, 9.5). Moreover, older medical students (≥23 years) had more accurate knowledge regarding diagnosis (β = 3.9, 95% CI: 1.8, 6.1) and higher total scores (β = 2.8, 95% CI: 1.1, 4.6). In addition, medical students who considered themselves not at risk for TB were associated with greater knowledge about treatment (β = − 3.5, 95% CI: -6.5, − 0.6).

**Table 5:** Multiple linear regression analysis of determinants of the percentage of correct answers

	β	*S*_*b*_	b’	t	*P*	95%CI
Epidemiology and prevention						
constant	40.8	1.3		30.6	< 0.001	(38.2, 43.5)
Observed at least one TB case						
Yes	5.6	1.7	0.1	3.4	0.001	(2.4, 8.9)
Observed at least one X-ray of TB case						
Yes	8.2	1.8	0.2	4.5	< 0.001	(4.6, 11.8)
Diagnosis						
constant	24.8	1.4		17.8	< 0.001	(22.0, 27.5)
Age						
≥ 23	3.9	1.1	0.1	3.6	< 0.001	(1.8, 6.1)
Observed at least one TB case						
Yes	3.9	1.6	0.1	2.4	0.015	(0.8, 7.1)
Observed at least one X-ray of TB case						
Yes	5.7	1.8	0.1	3.2	0.001	(2.2, 9.2)
Treatment						
constant	34.2	1.8		18.7	< 0.001	(30.6, 37.8)
Observed at least one TB case						
Yes	10.1	2.3	0.2	4.5	< 0.001	(5.68, 14.5)
Observed at least one X-ray of TB case						
Yes	7.0	2.5	0.1	2.8	0.005	(2.2, 11.8)
I am at risk for TB						
Yes	−3.5	1.5	−0.1	−2.4	0.019	(−6.5, −0.6)
Total						
constant	32.1	1.1		28.3	< 0.001	(29.9, 34.4)
Age						
≥ 23	2.8	0.9	0.1	3.2	0.002	(1.1, 4. 6)
Observed at least one TB case						
Yes	5.5	1.3	0.2	4.2	< 0.001	(2.9, 8.1)
Observed at least one X-ray of TB case						
Yes	6.7	1.5	0.2	4.6	< 0.001	(3.8, 9.5)

## Discussion

We found that medical students had generally poor knowledge about TB. More than 50% of the total questions regarding TB knowledge were incorrect, which was consistent with another survey conducted in southwest China in 2011 [16] that reported that fewer than half the students had knowledge of TB symptoms of cough/blood-tinged sputum, local TB dispensaries and the national free TB treatment policy. Compared with the results reported by Laurenti [19], the total mean percentage of correct answers in our study was lower (44.4% vs 56.5%), as were those for epidemiology and prevention (52.5% vs 63.5%) and diagnosis (35.7% vs 54.1%); however, our results regarding treatment were slightly higher (47.5% vs 45.7%). Moreover, the general knowledge level for TB in the present study was inferior to other surveys conducted among medical students in Italy [11], Brazil [13], and National TB Curriculum Consortium (NTCC) schools [20], which suggested a considerable need for improvement in TB knowledge among medical students. Because this lack of knowledge may be an influencing factor for a higher TB prevalence, improved instruction in TB for medical students is necessary.

Medical students’ attitudes towards TB may play an integral role in how they manage TB patients during their careers [21]. However, our study indicated that the majority of medical students lacked an accurate TB attitude. Approximately one-quarter of the medical students considered themselves at risk of contracting TB, which was consistent with the results reported by Bhandari [22]. Nevertheless, in the subsequent multiple linear regression analysis, we observed a negative association between attitude and the level of knowledge of TB treatment. In other words, the medical students who believed they were not at risk of contracting TB had a higher level of knowledge of TB treatment. This finding disclosed that the medical students in our study lacked prevention awareness, although they had comparatively more TB knowledge. Therefore, we could infer that a relationship did not necessarily exist between knowledge and attitudes for TB and that higher levels of knowledge did not result in positive attitudes. However, this result might have been influenced by the sample size and questions asked. Admittedly, correct knowledge and positive perceptions towards TB were prerequisites to seek early prevention and control measures.

In our study, more than 80% of the medical students had observed at least one TB case and an X-ray of a TB patient; these students reflected a better grasp of TB knowledge. Furthermore, the multivariable linear regression analysis showed a significant association between observing at least one TB case and an X-ray of a TB patient and greater knowledge of epidemiology and prevention, diagnosis, and treatment and the total score. This finding suggested that clinical experience was important for promoting TB knowledge among medical students. This result might be related to the amount of curriculum time devoted to TB and the number of TB patients seen by the students [20, 23]. However, Hoffman [24] reported in 2016 that even among health professionals, a significant gap remained for improvement of knowledge and practices regarding TB.

We also found that older medical students (≥23 years) had more accurate TB knowledge about the diagnosis and total scores, leading to a positive association between age and the level of TB knowledge. Similarly, a study by Montagna [11] reported that correct answers to questions concerning TB and its vaccine were associated with increasing age. TB knowledge increased with age, probably because older medical students developed more correct attitudes and behaviours regarding the disease. The TB course schedule might also be responsible for this finding.

To appropriately address the specialized problems of TB prevention and control, education and competence need to be strengthened among health care providers. The U.S. National Institutes of Health (NIH) funded the NTCC in 2003 to develop educational programmes to improve TB knowledge and practices among health professions students [20]. China also published the National Guideline for TB control, which emphasized TB health education [25]. Compared to traditional teaching, modern training methods, such as student-based teaching methods (problem-based learning), lead to improved student satisfaction and provide additional learning opportunities [26]. Hence, the results of the present study highlight a demand to innovate modern educational methods to promote knowledge and social responsibility among medical students.

### Limitations

Our study has several limitations. First, the questionnaire from Laurenti may not be comprehensive and appropriately adapted to the specific situation in China. Second, the representativeness of the sample needs to be improved further by increasing the sample size or adopting randomized sampling methods. Third, gauging a person’s attitude towards TB based solely on the question “I am at risk for TB” is insufficient. This issue should be improved in a future study. Fourth, due to the cross-sectional study design, the causal relationship between the TB knowledge level and determinants could not be explored; however, the findings provide a basis for acquiring and testing a causal hypothesis. Finally, some reporting bias may have been inherent in this study design.

## Conclusion

The present study found poor knowledge of TB among final-year medical students and considerable room for improvement in terms of TB knowledge, attitudes and practices. Moreover, practical clinical experience, attitude and age were significantly associated with more TB knowledge through a multiple linear regression analysis. We present the current status of TB knowledge levels among medical students and promote the need for active-learning methodologies.

## Abbreviations

BCG: Bacille Calmette-Guerin
CI: Confidence interval
MDGs: Millennium Development Goals
PPD: Purified protein derivative
SD: Standard deviation
TB: Tuberculosis
WHO: World Health Organization

## References

[1] World Health Organization (2006). Available at: WHO/HTM/STB/2006.35. Last access February 2014. Stop TB partnership and the World Health Organization. The global plan to stop tuberculosis, 2006–2015.

[2] MC R, Uplekar MW (2006). WHO’s new stop TB strategy. Lancet.

[3] Dye C, Maher D, Weil D (2006). Targets for global tuberculosis control. Int J Tuberc Lung Dis..

[4] World Health Organization. WHO Global tuberculosis report 2015. Available from: URL: http://www.who.int/tb/publications/2015/en/. Accessed 21 Apr 2018.

[5] World Health Organization. WHO Global tuberculosis report 2017. Available from: URL: http://www.who.int/tb/publications/global_report/en/. Accessed 15 Apr 2018.

[6] Wang L, Zhang H, Ruan Y (2014). Tuberculosis prevalence in China, 1990-2010; a longitudinal analysis of national survey data. Lancet.

[7] Dye C, Lönnroth K, Jaramillo E (2009). Trends in tuberculosis incidence and their determinants in 134 countries. Bull World Health Organ.

[8] Woith WM, Volchenkov G, Larson JL (2010). Russian health care workers’ knowledge of tuberculosis and infection control. Int J Tuberc Lung Dis.

[9] Doosti Irani A, Hashemi Shahraki A, Ghaderi E (2015). Lack of optimum practice among health care workers regarding tuberculosis in Iran: a knowledge, attitude, and practice study. Am J Infect Control.

[10] Li Y, Ehiri J, Tang S (2013). Factors associated with patient, and diagnostic delays in Chinese TB patients: a systematic review and meta-analysis. BMC Med.

[11] Montagna MT, Napoli C, Tafuri S (2014). Knowledge about tuberculosis among undergraduate health care students in 15 Italian universities: a cross-sectional study. BMC Public Health.

[12] Mehta D, Bassi R, Singh M, Mehta C (2012). To study the knowledge about tuberculosis management and national tuberculosis program among medical students and aspiring doctors in a high tubercular endemic country. Ann Trop Med Public Health.

[13] Teixeira EG, Menzies D, Cunha AJ (2008). Knowledge and practices of medical students to prevent tuberculosis transmission in Rio de Janeiro, Brazil. Rev Panam Salud Publica.

[14] Behnaz F, Mohammadzade G, Mousavi-E-Roknabadi RS (2014). Assessment of knowledge, attitudes and practices regarding tuberculosis among final year students in Yazd, Central Iran. J Epidemiol Glob Health.

[15] Olakunle OS, Oladimeji O, Olalekan AW (2014). Knowledge of tuberculosis management using directly observed treatment short course therapy among final year medical students in south Western Nigeria. Pan Afr Med J.

[16] Zhao Y, Ehiri J, Li D (2013). A survey of TB knowledge among medical students in Southwest China: is the information reaching the target?. BMJ Open.

[17] Bai LQ, Xiao SY, Xie HW (2003). Knowledge and practice regarding tuberculosis among final year medical students in Hunan, China. Chin J Tuberc Respir Dis.

[18] Chen W, Xia YY, Li T (2016). Analysis for the GiobaI and China TB epidemic situation in 2015. J Tuberc Lung Health.

[19] Laurenti P, Federico B, Raponi M (2013). Knowledge, experiences, and attitudes of medical students in Rome about tuberculosis. Med Sci Monit.

[20] Jackson M, Harrity S, Hoffman H (2007). A survey of health professions students for knowledge, attitudes, and confidence about tuberculosis, 2005. BMC Public Health.

[21] Emili J, Scott F, Upshur RE (2002). Attitudes toward tuberculosis of final year medical students from Canada, India, and Uganda. Teach Learn Med.

[22] Sunita R, Bhandari RB (2016). Knowledge, attitude and practice against tuberculosis infection control among medical students and nursing staff. J Cont Med A Dent.

[23] Emili J, Norman GR, Upshur REG (2001). Knowledge and practices regarding tuberculosis: a survey of final-year medical students from Canada, India and Uganda. Med Educ.

[24] Hoffman SJ, Guindon GE, Lavis JN (2016). Surveying the knowledge and practices of health professionals in China, India, Iran, and Mexico on treating tuberculosis. Am J Trop Med Hyg.

[25] Center for Disease Control in China (2008). The operational guideline for tuberculosis control program in China.

[26] Ghasemzadeh I, Aghamolaei T, Hosseini-Parandar F (2015). Evaluation of medical students of teacher-based and student-based teaching methods in infectious diseases course. J Med Life.

